# Geographical Origin Traceability of Navel Oranges Based on Near-Infrared Spectroscopy Combined with Deep Learning

**DOI:** 10.3390/foods14030484

**Published:** 2025-02-03

**Authors:** Yue Li, Zhong Ren, Chunyan Zhao, Gaoqiang Liang

**Affiliations:** 1 Key Laboratory of Advanced Electronic Materials and Devices of Jiangxi Province, Jiangxi Science and Technology Normal University, Nanchang 330038, China; 2023010326@jxstnu.edu.cn (Y.L.); 2023010317@jxstnu.edu.cn (C.Z.); 15003869603@163.com (G.L.); 2 Key Laboratory of Optic-Electronic Detection and Information Processing of Nanchang City, Jiangxi Science and Technology Normal University, Nanchang 330038, China

**Keywords:** navel oranges, near-infrared spectroscopy, geographic origin traceability, deep learning, spectral preprocessing

## Abstract

The quality and price of navel oranges vary depending on their geographical origin, thus providing a financial incentive for origin fraud. To prevent this phenomenon, it is necessary to explore a fast, non-destructive, and precise method for tracing the origin of navel oranges. In this study, a total of 490 Newhall navel oranges were selected from five major production regions in China, and the diffuse reflectance near-infrared spectrum in 4000–10,000 cm^−1^ were non-invasively collected. We examined seven preprocessing techniques for the spectra, including Savitzky–Golay (SG) smoothing, first derivative (FD), multiplicative scattering correction (MSC), combinations of SG with MSC (SG+MSC), SG with FD (SG+FD), MSC with FD (MSC+FD), and three combined (SG+MSC+FD). A one-dimensional convolutional neural network (1DCNN) deep learning model for geographical origin tracing of navel orange was established, and five machine learning algorithms, i.e., partial least squares discriminant analysis (PLS-DA), linear discriminant analysis (LDA), support vector machine (SVM), random forest (RF), and back-propagation neural network (BPNN), were compared with 1DCNN. The results show that the 1DCNN model based on the SG+FD preprocessing method achieved the optimal performance for the testing set, with prediction accuracy, precision, recall, and F1-score of 97.92%, 98%, 97.95%, and 97.90%, respectively. Therefore, NIRS combined with deep learning has a significant research and application value in the rapid, nondestructive, and accurate geographical origin traceability of agricultural products.

## 1. Introduction

China is one of the leading production countries of navel oranges in the world. In China, regions suitable for navel orange cultivation are extensive, mainly distributed in the Central, Southeastern, and Southwestern regions of the country. For example, Ganzhou city in Jiangxi Province is one of the primary production areas of navel oranges, covering an area of 110,000 hectares and yielding an annual output of 1.2 million tons [1]. Navel oranges are widely favored due to their rich nutritional value and health benefits with high vitamin C, carotenoids, citric acid, cellulose, as well as trace elements such as magnesium, zinc, calcium, and iron, which can reduce the risk of heart disease, various cancers, and respiratory system diseases [2]. However, due to differences in growing environments such as temperature, rainfall, sunlight, and soil nutrients, there is significant variability in fruit quality among different production areas [3]. Generally, product quality determines market price. However, to seek high profits for some businesses, there are illegal sales of fake origin in the navel orange market. To protect the consumer rights, brand reputation, and regulate the sales market, it is necessary to perform the high accurate geographical origin traceability of navel oranges. In the past, the traditional method relying on human sensory evaluation involving the color, shape, and odor to identify the origin of navel oranges was highly subjective, resulting in a high error rate in identifying the origin of navel oranges and leading to low efficiency in manual identification. Therefore, the development of geographical origin traceability technology for navel oranges is essential [4].

In recent years, researchers have performed some valuable studies on the geographical origin identification of fruit and vegetable products. Wen et al. [5] utilized gas chromatography-mass (GC-MS) to distinguish the origin of winter jujubes and achieved an accuracy rate of 97.6%. Muñoz-Redondo et al. [6] applied multi-element and stable isotope characterization for authenticating the origin of commercial avocados and achieved an accuracy of 98% in distinguishing whether the avocados were of Spanish origin. Although these chemical analysis techniques for origin identification have achieved high accuracy, they are associated with high equipment costs, complex sample preparation, time-consuming sampling, and are destructive to the samples. Therefore, to explore a rapid, non-destructive, and accurate method for tracing the origin of navel oranges, a non-invasive method for the geographical origin traceability of navel oranges was studied in this work.

For the near-infrared spectroscopy (NIRS) method, the samples can be directly detected instead of complex sample preprocessing like the chemical method. At the same time, the price of NIRS spectrometers is cheaper than that of electronic sensors and chemical analysis instruments. NIRS is a kind of non-invasive, high efficiency, high performance-cost ratio, and convenient detection method. Currently, NIRS analysis technology has been applied in the research field of food origin traceability. Chanachot et al. [7] utilized NIRS to classify the geographical origin of durians and achieved a 94.7% accuracy rate. Schütz et al. [8] analyzed grain corn from five different countries using Fourier-transform near-infrared (FT-NIR) spectroscopy and achieved an average accuracy rate of 95% for origin identification. Wu et al. [9] utilized NIRS technology to authenticate the origin of Schisandra chinensis, and a classification accuracy rate of 97.47% was achieved. Similarly, Zhang et al. [10] employed NIRS for the origin traceability study of white tea (white peony), and an accuracy rate of 97.96% was reached. Moreover, Chen et al. [11] also applied NIRS to identify the geographical origin of ginseng and attained an accuracy rate of 99.3%. Hu et al. [12] employed NIRS to detect the origin of jujube, and the accuracy was 98.8%. Li et al. [13] used NIRS to identify the origin of Pinus Koraiensis seeds, and the accuracy of the calibration and prediction sets was 98.75% and 97.50%, respectively. These studies indicate that NIRS has the potential values for rapid, accurate, and non-destructive identification of the origin of foods. However, there are no reports about the researches and applications of NIRS in identifying origins of navel orange. Especially for the precise geographical origin traceability of navel oranges from multiple neighboring production areas of the same cultivar, ensuring a high identification accuracy rate is a challenge.

In the past few years, various machine learning methods have been developed to solve different types of data and problems [14]. However, traditional machine learning methods (e.g., PLS, KNN, SVM, RF) usually rely on structured data and have a certain limitation in the accuracy, generalization ability, and computing capacity to handling large data sets, and even suffer from the dimensionality curse. In contrast, deep learning networks have many hidden layers (such as convolutional layers) that are trained end-to-end to learn feature patterns and are suitable for larger-scale data [15]. In small-scale data, regularization and dropout layers can solve the overfitting problem of the network, and deep learning can perform better than machine learning methods [16]. In recent years, deep learning has achieved some accomplishments in the field of NIRS analysis technology. Xia et al. [17] utilized NIRS combined with convolutional neural networks (CNN) for plastic discrimination and attained an accuracy rate of 98%, while the accuracy rate of traditional machine learning algorithms was only 57% to 70%. Dong et al. [18] employed a CNN with attention mechanism and NIRS for the classification of mango varieties, and an accuracy of 98.67% was achieved. Chen et al. [19] constructed a one-dimensional convolutional neural network (1DCNN) based on NIRS data to achieve the classification of mixed fish and achieved an accuracy rate of 98%. Jiang et al. [20] utilized NIRS and two-dimensional convolutional neural network (2DCNN) to quantitatively analyze the aflatoxin B_1_ in moldy peanuts, and achieved a root mean square error (RMSE) of 2.0 μg∙kg^−1^ and a determination coefficient ($R^{2}$) of 0.99 for the prediction set. Wang et al. [21] used NIRS data to establish a lightweight convolutional neural network for nicotine prediction in tobacco. The RMSE was 0.14, and $R^{2}$ was 0.95.

Compared with traditional statistical methods and traditional machine learning models, deep neural networks including the 1DCNN model have some prominent advantages, such as a strong capacity to extract the characteristic information, higher efficiency and strong generalization, a strong local perception ability, and a strong ability to handle massive amounts of data. Up to now, the one-dimensional convolutional neural network (1DCNN) model has already been studied in various fields of foods and crops, including geographical origin traceability. For example, Ma [22] employed visible-near infrared spectroscopy (Vis-NIRS) combined with the 1DCNN model to identify the origin and predict the physiologically active ingredient contents of *Gastrodia elata* Blume. Their study has demonstrated that the 1DCNN model outperforms the other three machine learning algorithms due to the highly accurate non-linear descriptive capability. Jiang [23] utilized visible-near infrared (Vis-NIR) hyperspectral imaging technology combined with the 1DCNN model to discriminate different geographical origins of wolfberries, achieving an accuracy of 91.99%. Yang [24] employed NIRS combined with the 1DCNN model to perform the geographical traceability of American ginseng, with 98.95% accuracy, which outperformed the other four methods. Li et al. [25] used NIRS combined with the 1DCNN model to identify the origin traceability of jujubes from different production areas in Xinjiang, China, with accuracy of 90.43% for 700 samples. As evidenced by these studies, there is a keen interest within the scientific community in employing deep learning models for modeling purposes, and the application of deep learning in the field of NIRS is also booming [26], which provides a feasibility support for using NIRS combined with deep learning to achieve high-accuracy geographical origin traceability of navel oranges.

**Figure 1:**
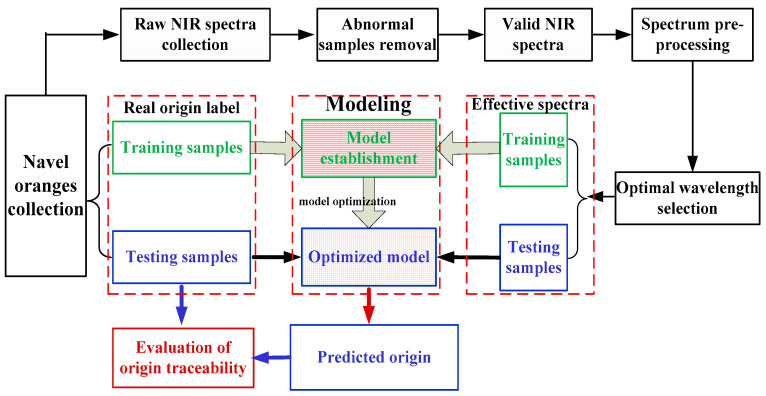
Flow chart of geographical origin traceability for navel oranges via NIRS combined with deep learning.

In order to achieve rapid, accurate, and non-destructive geographical origin traceability of navel oranges, the main work of this study is shown as follows: (1) Perform the experiments for collecting NIRS data of navel orange samples sourced from different origins via FT-NIR spectrometer. (2) Investigate and compare the results of various preprocessing methods (both individual and combined methods) based on the partial least squares discriminant analysis (PLS-DA) model, and determine the preprocessing technique most suitable for the origins traceability of navel oranges. (3) Construct a 1DCNN model for geographical origin traceability of navel oranges and optimize the model parameters. (4) Compare the origins traceability performance of navel oranges based on the 1DCNN established in this study with the traditional machine learning algorithms. The aim of this study is to provide a comprehensive and systematic way of geographical origin traceability and its applicability for navel oranges by using NIRS combined with deep learning. The flow chart of geographical origin traceability for navel oranges via NIRS combined with deep learning is presented in Figure 1.

## 2. Materials and Methods

### 2.1. Sample Preparation

**Figure 2:**
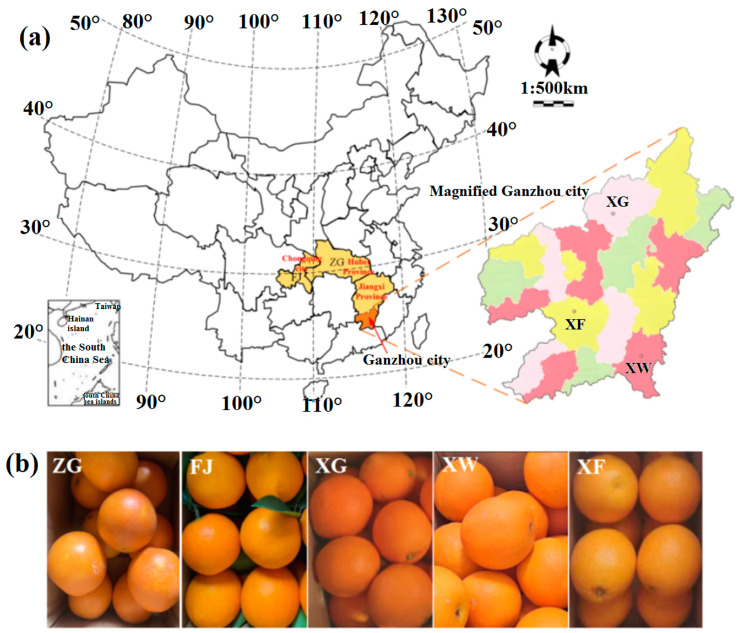
(**a**) The geographical distribution of the navel orange samples from five origins in China; (**b**) photos of the navel orange samples.

In this study, to avoid the impact of variety differences and season harvest on the origin traceability of navel oranges, 490 mature Newhall navel oranges were synchronously harvested in 15–16 December 2023 from five different country-towns, i.e., Xingguo (XG, *n* = 100), Xunwu (XW, *n* = 100), and Xinfeng (XF, *n* = 100) in Ganzhou city of Jiangxi province, Zigui (ZG, *n* = 94) in Hubei province, and Fengjie (FJ, *n* = 96) in Chongqing city. Since the Southern region of Ganzhou city in Jiangxi province is the top navel orange-producing area in China, there are several planting areas located in the Southern region of Ganzhou city. To ensure the high accuracy origin traceability of navel oranges, three country-towns in Ganzhou city were selected in the same province. Figure 2a shows the geographical distribution of the navel orange samples. Five production areas are concentrated in the central region of China, i.e., Zigui county-town in Yichang city of Hubei province (30°49′39.3″ N, 110°58′21.1″ E), the average annual precipitations and average annual sunshine duration are 1016 mm and 1652 h, the average annual temperature is 17.6 °C; Fengjie county-town in Chongqing city (31°1′23.8″ N, 109°24′20.2″ E), the average annual precipitations and average annual sunshine duration in Fengjie are 1021 mm and 1341 h, the average annual temperature is 18.7 °C; three county-towns in Ganzhou city of Jiangxi province, i.e., Xingguo county-town (26°20′28.4″ N, 115°21′28.7″ E), the average annual precipitations and average annual sunshine duration are 1074.6 mm and 1926.5 h, the average annual temperature is 18.8 °C; Xinfeng county-town (25°23′19.9″ N, 114°55′6.0″ E), the average annual precipitations and average annual sunshine duration are 953.9 mm and 1700 h, the average annual temperature is 20.2 °C; and Xunwu county-town (24°58′17.9″ N, 115°38′5.3″ E), the average annual precipitations and average annual sunshine duration are 1616.8 mm and 1823.8 h, the average annual temperature is 20.3 °C. In each production area of navel oranges, the samples were harvested from the same orchard. In addition, to avoid the impact of fertilizer on the origin traceability of navel oranges, during the planting of navel oranges in different origins, the trees of navel oranges were fertilized using the same fertilizers, such as animal manure organic fertilizer, nitrogen fertilizer, phosphorus fertilizer, and potassium fertilizer.

Figure 2b presents photos of the navel orange samples sourced from the aforementioned five areas. Although the size, color, and texture of the navel oranges from five different areas appear similar, variations in growth environment, climate, and soil among these geographical origins contribute to differences in taste and nutritional content of navel oranges.

Before the experiment, all navel oranges were cleaned with clean water to remove surface stains, then dried using absorbent paper. The surface moisture was further evaporated by leaving them in a well-ventilated area for 24 h. Finally, each navel orange was numbered according to its origin.

### 2.2. Spectra Acquisition

**Figure 3:**
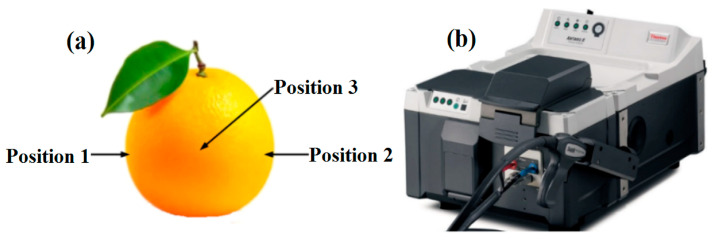
(**a**) Spectra collection positions of the navel orange samples; (**b**) FT-NIR spectrometer.

Near-infrared spectra of all navel orange samples were collected using a Fourier transform near-infrared spectrometer (Antaris™ II FT-NIR, Thermo Fisher Scientific, Waltham, MA, USA) with an integrating sphere diffuse reflectance module, as shown in Figure 3b. The software employed for collecting the near-infrared spectral data was Thermo RESULT Integration (Thermo Fisher Scientific, Waltham, MA, USA). The experimental environmental temperature was controlled at 22 ± 0.5 °C. Spectra acquisition parameters were set, e.g., wave-number range of 4000–10,000 cm^−1^, scanning times of 64, and spectral resolution of 8.0 cm^−1^.

To ensure the reliability of NIR spectral data collected from navel oranges, the FT-NIR spectrometer was preheated for 30 min before the experiment. Before collecting the spectra of samples, the air background spectra was deducted to reduce the impact of environmental factors on the spectral data. During the collection of NIR spectra, the navel orange samples were placed on the sample holder of the integrating sphere diffuse reflection module. The spectra of three different positions on the equatorial region of the navel oranges were collected as shown in Figure 3a, and the average spectra were used as the original spectral data for the navel orange.

### 2.3. Removal of Abnormal Samples

When collecting NIR spectral data of samples, abnormal data may be generated due to the effect of instrumental and environmental noises. To reduce the impact of abnormal data on the modeling effect, removal of abnormal samples is required before modeling. In this study, the Mahalanobis distance method [27] was used to remove abnormal samples. The Mahalanobis distance helps identify and remove abnormal spectra that do not conform to the overall data distribution by quantifying the differences between spectral data. After removal of abnormal samples, there were 478 samples. The number of navel oranges sourced from each production area before and after removal is shown in Table 1. To establish the calibration model, 478 normal samples were randomly divided into the training set and testing set in an 8:2 ratio, i.e., 382 samples were used as the training set and 96 samples were used as the testing set. Moreover, the training subset and testing subset are independent of each other; that is, there are no identical samples in the training and testing sets.

### 2.4. Spectra Pretreatment

During the process of collecting NIR spectra in experiments, the collected NIR spectra not only contains information related to navel orange samples, but also is contaminated with interfering information, and is influenced by factors such as random noise, background interference, and light scattering. To reduce the influence of interfering information on the modeling effect, several types of preprocessing methods for spectral preprocessing were selected, e.g., Savitzky–Golay smoothing (SG) [28], multiplicative scattering correction (MSC) [29] and first derivative (FD) [30]. SG is usually utilized to remove noise and enhance the signal-to-noise ratio of spectra. MSC is usually employed to eliminate the scattering effect of light in the NIR spectra. FD is usually utilized to highlight the peaks and troughs in the spectra, subtract the background from the spectrum, and eliminate the baseline shift of the original spectra. To explore the impact of different preprocessing methods, eight spectra preprocessing methods, i.e., no preprocessing (No-preprocess), single preprocessing methods (SG, MSC, and FD), and combinations of multiple preprocessing methods including SG combined with MSC (SG+MSC), SG combined with FD (SG+FD), MSC combined with FD (MSC+FD), and SG combined with MSC and FD (SG+MSC+FD), were applied to the raw spectra and compared with one another. Partial least squares discriminant analysis (PLS-DA) models [31] were employed to evaluate the performance of each spectra preprocessing method, and the optimal spectra preprocessing method was determined based on RMSE and R^2^. The comparisons of spectra preprocessing methods were implemented in Python v3.9.7.

### 2.5. Establishment of Models

In order to establish the connection between navel orange spectra and origin information, the preprocessed spectral data were used to establish a one-dimensional convolutional neural network (1DCNN) [32] model to classify the origins of navel orange samples. At the same time, PLS-DA, linear discriminant analysis (LDA) [33], support vector machine (SVM) [34], random forest (RF) [35], and back-propagation neural network (BPNN) [36] were compared with the 1DCNN model. Five different origins of navel oranges were labeled as ‘0’, ‘1’, ‘2’, ‘3’, and ‘4’ for XG, XF, XW, FJ, and ZG, respectively. Then, the NIR spectra and labels of navel oranges were used to establish classification models of geographical origin traceability of navel oranges in the training set. All models were implemented in Python v3.9.7.

#### 2.5.1. Traditional Machine Learning Models

PLS-DA [37] combines partial least squares (PLS) regression with discriminant analysis (DA), which is primarily used for classification problems, especially in high-dimensional data. Specifically, PLS-DA first establishes a PLS regression model between the labels (*Y*) and the spectral data (*X*). By maximizing the covariance between *X* and *Y*, a series of latent variables are extracted. Discriminant analysis and classification are then performed based on these latent variables. In the process of constructing the PLS-DA model, a 10-fold cross-validation method [38] was employed in this study to calculate the cross-validation accuracy for different numbers of latent variables. Finally, the number that yielded the highest cross-validation accuracy was selected as the optimal number of latent variables for the PLS-DA model.

LDA [39] is a classical statistical and machine learning method primarily used for classification tasks [40]. Similarly, a 10-fold cross-validation method was employed to calculate the cross-validation accuracy rates under different principal components (PCs), and the optimal number of PCs for LDA was selected as the one yielding the highest cross-validation accuracy.

SVM is a pattern-recognition method proposed based on statistical principles [41], which exhibits advantages in addressing pattern-recognition problems with small samples, nonlinearity, and high dimensionality. The core idea of SVM lies in the result risk-minimization principle of statistical theory. Through kernel function transformation, the samples that are linearly inseparable in the low-dimensional space are mapped onto a high-dimensional space, where a classification hyperplane that maximizes the inter-class distance is identified, thereby fulfilling the purpose of classification. There are three optimizable hyperparameters in the SVM model, i.e., the kernel function (kernel), the penalty coefficient (*C*), and the kernel function parameter (γ). In this study, the grid search method was employed to seek the optimal combination of hyperparameters for the SVM model.

RF is an ensemble learning approach that conducts predictions by constructing multiple decision trees [42]. Each tree is trained with a random subset of the data and a random subset of the features. The ultimate prediction outcome is the majority vote of all the decision trees’ prediction results. This approach can reduce the risk of overfitting and enhance the stability and accuracy of the model. The modeling effect of RF is influenced by hyperparameter settings including the number of decision trees (n_estimators), the maximum depth of the trees (max_depth), the minimum number of samples required to split an internal node (min_samples_split), the minimum number of samples at leaf nodes (min_samples_leaf), and the maximum number of features for each split (max_features). The optimal parameter combination of RF is also sought using the grid search method.

BPNN is a classical artificial neural network model based on the multilayer perceptron (MLP) [43]. It can perform complex nonlinear mappings through multiple hidden nodes. It consists of the input layer, hidden layers, and the output layer. The training process of BPNN involves two key phases, i.e., forward propagation and backward propagation. During forward propagation, input data is transmitted through the network to the output layer, while in backward propagation, the network parameters are adjusted based on loss function to minimize prediction error.

#### 2.5.2. DCNN

1DCNN is a deep convolutional neural network model that specializes in processing one-dimensional data and has broad application prospects in one-dimensional spectral data processing [24]. The architecture of 1DCNN mainly includes input layer, one-dimensional convolution layer, activation function layer, pooling layer, fully connected layer, and output layer. Although 1DCNN has broad application prospects in one-dimensional data processing, the parameter adjustment problem of CNN is a very important topic due to its complexity and importance. For deep neural networks, their structures are generally complicated due to the multiple layers; the layer numbers of some deep models reach dozens, even hundreds. Moreover, there are a large number of parameters in the deep neural networks, such as the kernel size, kernel number, slide step, pooling size, batch size, dropout rate, learning rate, weights, biases, and activation function. For some deep neural networks, the parameters number can reach hundreds of thousands, even millions. At the same time, the layer number and parameters will determine the performance of the deep neural network. Due to the complicated structure and huge parameters, the excellent performance of the CNN model can generally be obtained through a reasonable model structure and parameters adjustment. It is worth mentioning that some experience and skill are also crucial for the tuning efficiency of deep learning models. In this work, to obtain the satisfactory origin traceability of navel oranges, the structure and parameter adjustment of the 1DCNN model was investigated.

### 2.6. Evaluation of Models

To evaluate the performances of models, a variety of evaluation indicators [7] were employed in this study, i.e., accuracy (Acc), precision (P), recall rate (R), and F1 score (F1). In addition, to minimize the risk of overfitting the model on specific data and enhance the generalization ability of the model, the 10-fold cross-validation (CV) accuracy of the model was utilized in this study.

## 3. Results

### 3.1. Spectral Analysis

**Figure 4:**
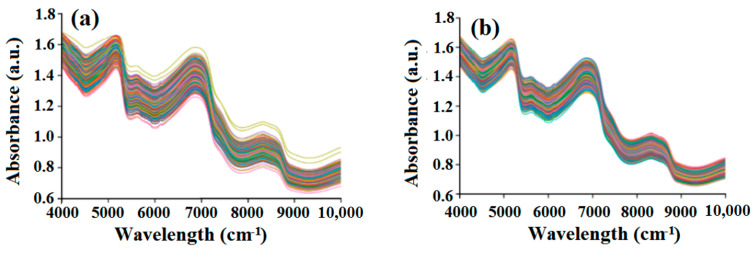

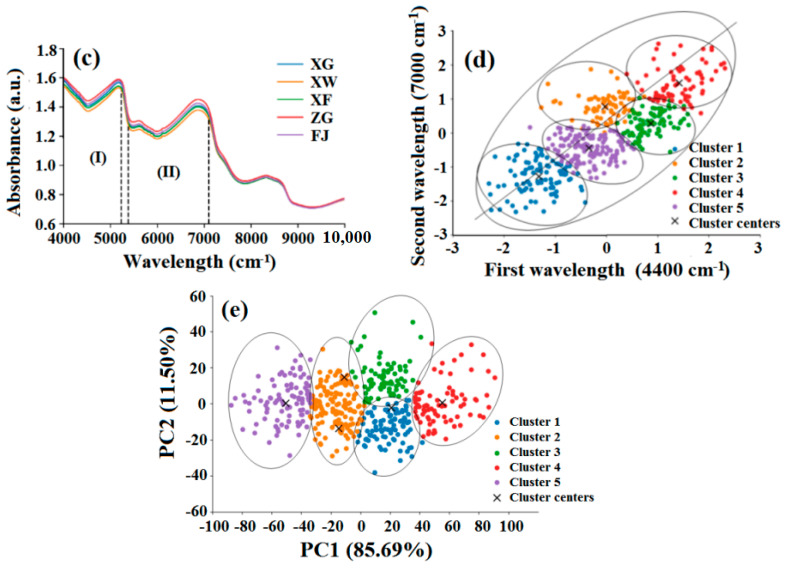
(**a**) NIR spectra of 490 samples before the removal; (**b**) NIR spectra of 478 samples after the removal; (**c**) The average NIR spectra of navel oranges from five origins; (**d**) The clustering results of navel oranges via the *k*-means algorithm; (**e**) The clustering results of navel oranges via the PCA algorithm.

Figure 4 presents the original NIR spectra of navel orange samples in the 4000–10,000 cm^−1^. Figure 4a shows NIR spectra of 490 samples before the removal based on the Mahalanobis distance, while Figure 4b displays those of 478 samples after the removal. From Figure 4a,b, it can be seen that the NIR spectra that significantly deviate from the other spectral curves are excluded, suggesting that the Mahalanobis distance to eliminate abnormal samples is reliable. Figure 4b shows the trend changes of navel orange spectra are basically consistent without any significant differences. With the increase of wavenumber, the absorbance shows an overall decreasing trend. As we know, the NIR spectra can reflect the first, second, and third overtones of O-H, C-H, S-H bands stretching vibrations and their combination, related with some physicochemical components. From Figure 4a, it can be seen that there were strong water-absorption peaks near 5200 cm^−1^, 6890 cm^−1^, and 8500 cm^−1^ [44]; absorption peaks of vitamin C in 4950~5790 cm^−1^ and 7800~8733 cm^−1^ [45]; absorption peaks of fructose in 6361~6369 cm^−1^ and 6527~6544 cm^−1^ [46]; absorption peaks of fruit acid near 6238 cm^−1^, 7223 cm^−1^, and 8700 cm^−1^ [47]; and absorption peaks of dry matter in 4255~4651 cm^−1^ and 5263~6666 cm^−1^ [48]. To identify the spectra differences of navel oranges from different origins, the average NIR spectra of navel oranges from five origins were plotted, as shown in Figure 4c. In the 4000–5230 cm^−1^ (see zone I in Figure 4c) and 5380–7100 cm^−1^ (see zone II in Figure 4c), the average spectra of navel oranges from different origins exhibit obvious differences. From top to bottom, they represent the origins of ZG, FJ, XG, XF, and XW, respectively. The reason can be explained as follows: Navel oranges from different origins contain varying contents of nutrient components, resulting in the distinctions in the NIR absorbance of navel oranges in the corresponding wavebands. Therefore, it is feasible to identify the origin of navel oranges using the NIRS with the “fingerprint effect”.

To further explore the differences in spectral data from various origins, the *k*-means algorithm [49] and principal component analysis (PCA) algorithm [50] were employed for unsupervised clustering of navel oranges based on the raw spectra. The clustering results based on *k*-means and PCA are shown in Figure 4d,e, respectively. In Figure 4d, the *x*- and *y*-axes represent wavelengths with significant average spectra differences in wavebands (I) and (II) of Figure 4c (4400 cm^−1^ and 7000 cm^−1^). In Figure 4e, the *x*- and *y*-axes represent the first and second principal components (PC1 and PC2) of PCA. Although the above two unsupervised clustering algorithms divide the NIR spectra of navel oranges into five clusters, determining the specific origin of each navel orange requires supervised learning algorithms.

### 3.2. Spectral Pretreatment

**Figure 5:**
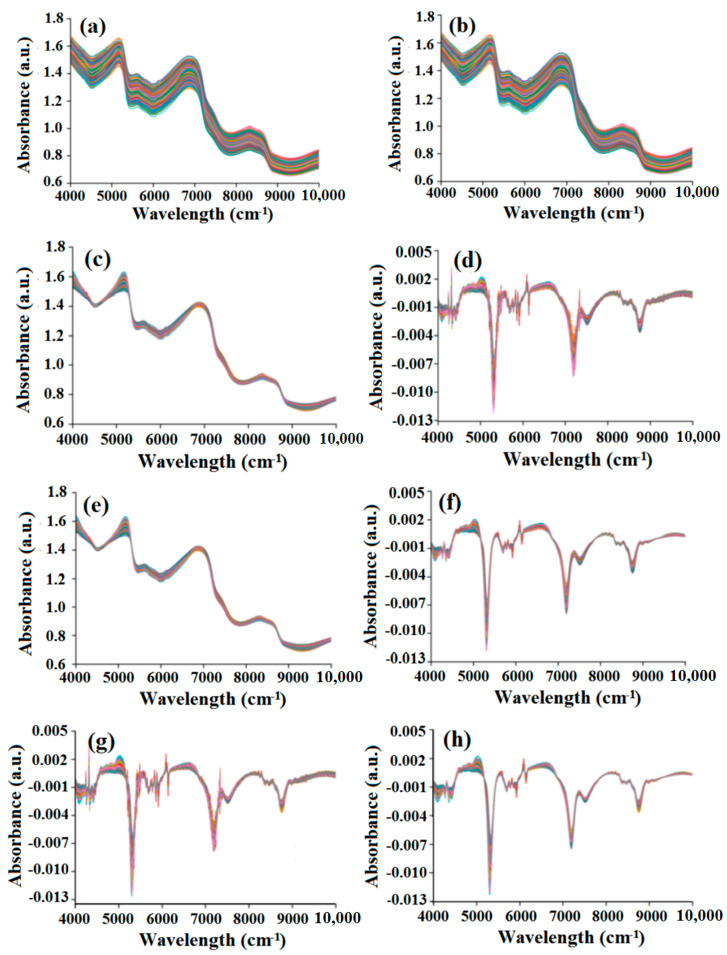
The preprocessed spectra via different pretreatment strategies. (**a**) No-preprocess, (**b**) SG, (**c**) MSC, (**d**) FD, (**e**) SG+MSC, (**f**) SG+FD, (**g**) MSC+FD, (**h**) SG+MSC+FD.

Although the raw NIR spectra of navel orange samples contain information about the origin of the navel oranges, baseline drift and peak overlap will influence the modeling effect of origin traceability. To achieve better traceability accuracy, it is necessary to preprocess the raw spectra. In this study, three preprocessing methods, i.e., SG, MSC, and FD, were selected. However, combining multiple preprocessing methods may be more helpful for modeling analysis. Therefore, eight preprocessing strategies including No-preprocess, SG, MSC, FD, SG+MSC, SG+FD, SG+MSC, and SG+MSC+FD, were employed. Figure 5 shows the preprocessed spectra via different strategies.

To compare different preprocessing strategies, the PLS-DA model was employed for the preprocessed spectra, and the optimal preprocessing strategy was determined based on the accuracy of the training set and the testing set. The optimal number of principal components (PCs) of the PLS-DA model was selected using the 10-fold cross-validation method. Table 2 shows the results of PLS-DA models with different preprocessing strategies.

It can be seen from Table 2 that the classification performance via SG+FD strategy was the best due to the highest accuracy. The accuracies of the training and testing sets were 89.79% and 84.38%, respectively. Compared with no pretreatment (Figure 5a), SG (Figure 5b), MSC (Figure 5c), and SG+MSC (Figure 5e), the SG+FD strategy (Figure 5f) could highlight the absorption peaks of the spectra. Compared with FD (Figure 5d) and MSC+FD (Figure 5g), the SG+FD strategy could effectively eliminate spikes in the navel orange spectra and make the spectra smoother. For the combination of three preprocessing methods, i.e., SG+MSC+FD, its classification accuracy was lower than SG+FD. Therefore, SG+FD strategy was utilized to preprocess the raw NIR spectra of navel oranges in this study.

### 3.3. Establishment of 1DCNN Model

**Figure 6:**
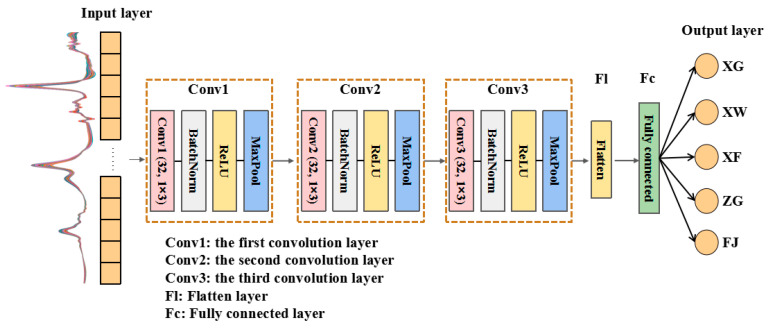
The established architecture of 1DCNN.

In this study, the structure of the 1DCNN model with multiple convolutional layers was constructed. The ReLU activation function was utilized after each convolutional layer. Compared with the sigmoid function, the ReLU function can effectively solve the problem of gradient disappearance and improve computational efficiency. A batch normalization (BatchNorm) layer was also added into the network to reduce internal covariate shift by normalizing the distribution of input data, thereby accelerating the convergence speed, improving the generalization ability of the network, and simplifying the network structure. The maximum pooling (MaxPool) was employed in the pooling layer, i.e., the largest value in the pooling field was selected, thereby reducing the data dimension and enhancing the representation ability of features. To obtain the optimal origin traceability of navel oranges based on the 1DCNN model, the effects of different convolution layer numbers, kernel size, batch size, and learning rate on the performance of the 1DCNN model were explored. By comparing the effect of the 1DCNN model with different parameters, a three-layer 1DCNN model was established in this study, which is shown in Figure 6.

The input data of the 1DCNN model is the NIR spectra of navel oranges. In three convolutional layers of the 1DCNN model, the structure of the convolutional kernel is also one-dimensional. In each convolutional layer, (32, 1 × 3) means that the convolutional kernel size of 1 × 3 is utilized, i.e., the row is 1 and the column is 3. The number of convolutional kernels is 32. After going through three convolutional layers, some useful information related to the geographical origin in the spectra are extracted. They are then flattened into a one-dimensional vector by the flatten layer and fed into the fully connected layer to realize origin identification. Since the navel oranges in this study come from five production areas, the output layer consists of five neurons. The output layer utilizes a softmax function to produce the classification probabilities for five origins of navel oranges, thereby achieving traceability of the geographical origin.

### 3.4. Parameter Optimization of the 1DCNN

**Figure 7:**
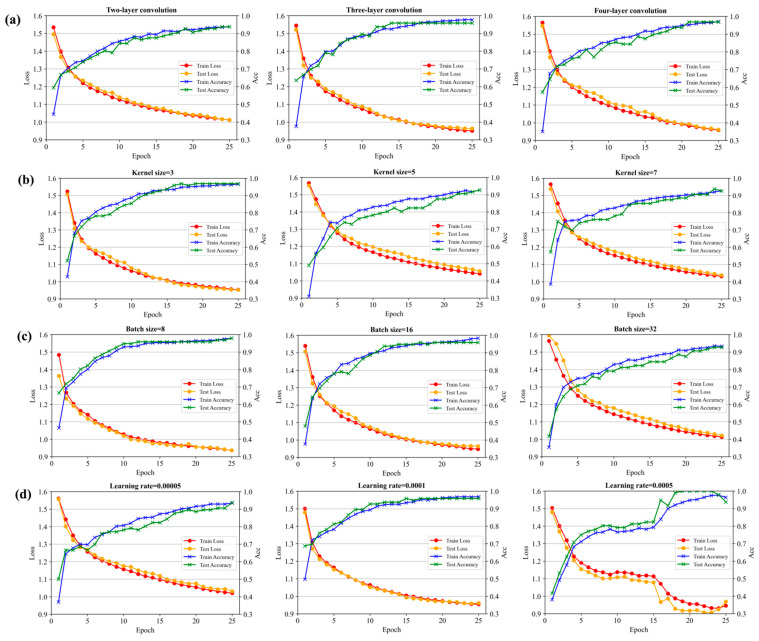
Accuracy curve and loss curve for different parameters of the 1DCNN model: (**a**) with different convolution layers; (**b**) with different kernel sizes; (**c**) with different batch sizes; (**d**) under different learning rates.

Compared with traditional machine learning algorithms, the 1DCNN involves more parameters, such as number of convolutional layers, kernel size, learning rate, and batch size. These parameters can significantly affect both the performance and computational efficiency of the model. To find the optimal structure and parameters of the model for the origin traceability of navel oranges, multiple structures of 1DCNN models were developed in this study. While the other parameters were kept the same, the influence of the number of convolutional layers, kernel size, batch size, and learning rate on model performance was investigated, respectively. The results are presented in Figure 7a–d.

Figure 7a shows the impact of the number of convolutional layers on the performance of the 1DCNN model. The number of convolutional layers directly affects the computational efficiency and speed of the model. Therefore, two-layer, three-layer, and four-layer convolutions were trained, respectively. When epoch was 25, the accuracy of two-layer convolution was about 94%, and the loss was greater than about 1; the accuracy of three-layer convolution and four-layer convolution was about 98%, and the loss was less than 1. Three-layer convolution and four-layer convolution had higher accuracy and lower loss, but when the results are similar, we tend to choose a simpler network structure, so a three-layer convolution 1DCNN model was chosen in this study.

Figure 7b presents the influence of the convolution kernel size on the performance of the 1DCNN model. In the convolution operation, the convolution kernel moves over the input data in a sliding window manner to complete the convolution computation. The size of the sliding window is kernel size, which determines the level of detail in feature extraction. Since the kernel size is often an odd number, kernel sizes of 3, 5, and 7 were compared in this study. When the epoch was 25, the accuracy for kernel size of 3 was approximately 98%, and the loss was 0.95; the accuracy for kernels sizes of 5 and 7 was around 92%, and the loss was higher than 1. Compared with kernels sizes of 5 and 7, the kernel size of 3 could extract more detailed features. Therefore, 3 was chosen as a value of kernel size in this study.

Figure 7c shows the impact of batch size on the performance of the DCNN model. Batch size refers to the number of samples trained by the model in each training iteration. A batch size that is too large may lead to insufficient model training, while a batch size that is too small may result in under-utilization of computational resources, thus reducing computational efficiency. Batch size is usually chosen as a power of 2. In this study, batch sizes of 8, 16, and 32 were compared. When the epoch was 25, the model achieved an accuracy of 92% with a batch size of 32, and the loss was greater than 1. For batch sizes of 8 and 16, the accuracy was around 98%, with loss less than 1. Both batch sizes of 8 and 16 achieved better training performance. However, the training time with a batch size of 8 was 11.8 s, while with a batch size of 16 was only 5.5 s. Considering both model performance and computational efficiency, a batch size of 16 was selected in this study.

Figure 7d shows the impact of learning rate on the performance of the 1DCNN model. In the training of deep learning models, the learning rate determines the size of the weight update during backpropagation. A too small learning rate may cause the model to learn very slowly, while a too large learning rate may lead to gradient explosion. Therefore, the effects of different learning rates (0.00005, 0.0001, and 0.0005) on the performance of the model were compared. When the epoch reached 25, the performance with a learning rate of 0.00005 was relatively poor, with an accuracy of 93%. Both learning rates of 0.0001 and 0.0005 achieved higher accuracy and lower loss. However, the performance of the model was more stable with the learning rate of 0.0001 than with 0.0005. Therefore, the learning rate of 0.0001 was selected in this study.

### 3.5. Comparison Between 1DCNN and Traditional Machine Learning Models

To effectively identify the origin of navel oranges, a 1DCNN model was established based on the optimal parameters aforementioned determined in this study. Meanwhile, multiple machine learning models (PLS-DA, LDA, SVM, RF, and BPNN) were employed to compare with the 1DCNN model. Table 3 presents the comparison of model performance for the origin traceability of navel oranges between 1DCNN and PLS-DA, LDA, SVM, RF, and BPNN.

From Table 3, it can be seen that the highest accuracy of the machine learning models on the testing set only reached 84.38%. The accuracy of the traditional machine learning algorithms for the training set, testing set, and 10-fold cross-validation was 77.75~89.79%, 69.79~84.38%, and 70.02~80.40%, respectively. For the 1DCNN model established in this study, the accuracy of the training set was 98.43%. The accuracy, precision, recall, and F1 score of the testing set based on the 1DCNN model were 97.92%, 98.00%, 97.95%, and 97.90%, respectively, and the accuracy of the 10-fold cross-validation was 97.50%. Compared with the traditional machine learning algorithms, it can be demonstrated that the performance of the 1DCNN achieved a significant improvement.

**Figure 8:**
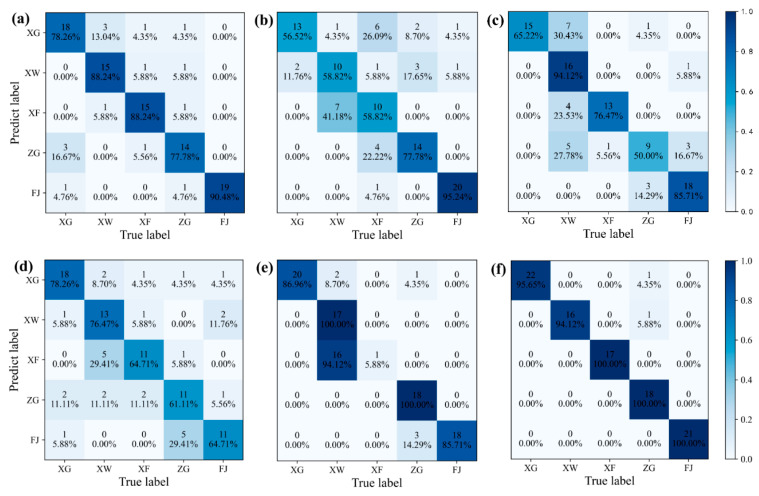
Confusion matrix for the different models. (**a**) PLS-DA; (**b**) LDA; (**c**) SVM; (**d**) RF; (**e**) BPNN; (**f**) 1DCNN.

To further visualize the performance of different models in the origin traceability of navel oranges, the confusion matrix [51] was employed this study. Generally, in the confusion matrix, a higher value on the diagonal and a darker color can indicate better classification performance of the model. Figure 8a–f shows the confusion matrices for PLS-DA, LDA, SVM, RF, BPNN, and 1DCNN, respectively. The predictive performance of PLS-DA for the five origins of navel oranges was moderate; the highest accuracy was 90.48% for FJ. For the LDA model, it performed poorly in predicting three origins in Ganzhou city of Jiangxi province (XG, XW, and XF), but showed high discrimination for FJ with an accuracy of 95.24%. For the SVM model, it had poor prediction for ZG with an accuracy rate of 50.00%, yet a classification accuracy of 94.12% for XW was achieved. For the RF model, it also had a moderate predictive ability for all five origins. For the BPNN model, its performance was extreme because the classification accuracies of XW and ZG were 100.00%, but the discrimination for XF was very poor with only one correct classification. However, for the 1DCNN model, it had good classification performance for all five origins, achieving 100.00% accuracy for XF, ZG, and FJ, and only one was misclassified for XG and XW. Therefore, compared with traditional machine learning models, the 1DCNN exhibited significant stability and high accuracy, and realized excellent origin traceability of navel oranges.

## 4. Discussion

### 4.1. The Effect of Wavelength Optimal Selection Algorithms on 1DCNN

Based on the established 1DCNN model, the influence of wavelength optimal selection on the origin traceability of navel oranges was investigated. In this study, five-wavelength optimal selection algorithms were employed, i.e., least angle regression (LAR) [52], competitive adaptive reweighted sampling (CARS) [53], uninformative variable elimination (UVE) [54], successive projections algorithm (SPA) [55], and genetic algorithm (GA) [56], and were compared with no-wavelength optimal selection (None). The spectral data selected by the five-wavelength optimal selection algorithms and the full spectra without wavelength optimal selection were respectively input into the 1DCNN model for the origin traceability of navel oranges. The comparison results are presented in Table 4.

It can be seen from Table 4, based on the 1DCNN model, the wavelength optimal selection method of SPA had the lowest accuracy; the testing set accuracy was less than 80%. Although the origin traceability of navel oranges based on GA had the highest accuracy with the testing set accuracy of 95.83%, it was still lower than the accuracy of no-wavelength optimal selection (97.92%). In general, although the wavelength optimal selection algorithm can improve the performance of the model in principle, in this work, the usage of the wavelength optimal selection algorithm reduced the accuracy of origin traceability for navel oranges. The reason may be that although the wavelength-optimization algorithms aim to remove redundant and irrelevant wavelengths, they may delete some important information that contribute significantly to model prediction of origin traceability of navel oranges, which results in the performance reduce of the classification model. However, the spectra with full wavelength retain the diversity of information to a certain extent, which is more helpful for the 1DCNN model to learn more important features. In addition, judging from the origin traceability time cost of different wavelength-optimal selection algorithms combined with the 1DCNN model, although the training and prediction time of modeling for no-wavelength optimal selection (None) was slightly longer than the time of modeling for LAR, CARS, and GA, the time cost of all wavelength-optimal selection methods was much greater than the time cost of model training and prediction. In particular, the time cost of GA was hundreds of times that of the modeling. Regarding the total time cost, the time of the 1DCNN model combined with the no-wavelength optimal selection (None) method was much smaller than the time of the 1DCNN model combed with other wavelength-optimal selection algorithms. For UVE and SPA, the limited number of selected wavelengths caused the model to spend longer training and prediction time of their models than that of the no-wavelength optimal selection method. Therefore, in this study, the no-wavelength optimal selection method (None) was employed. It was not only more helpful for 1DCNN modeling and achieved higher origin traceability accuracy of navel oranges, but it also reduced computing costs and improved computing efficiency.

### 4.2. Comparison Between NIRS and Machine Vision for Origin Traceability of Navel Orange

**Figure 9:**
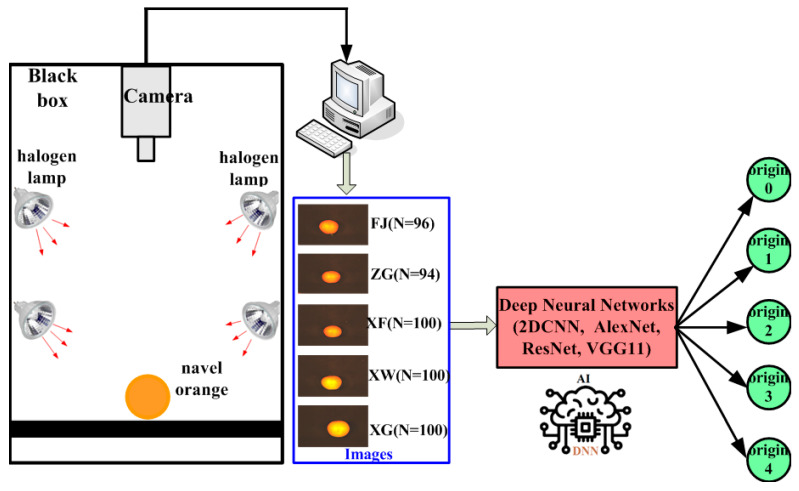
Schematic diagram of origin traceability of navel oranges by machine vision.

To validate the advantages of NIRS in geographical origin traceability of navel oranges, the machine vision technique [57] was also employed for origin traceability of navel oranges and compared with NIRS technology in this study. The RGB images of all navel orange samples were captured in experiments. The navel orange samples in machine vision experiments were the same with the samples in the NIRS experiments. As shown in Figure 9, a black box was constructed, and four halogen lamps (50 W) were installed on the four sides of the box to illuminate the navel oranges. The camera (Gaia Micro-V10-DZ, Suzhou, China) was employed to acquire images of all navel oranges, and the pixel size of each image was 2048 × 938. The image of navel oranges from five different origins were labeled as ‘0’, ‘1’, ‘2’, ‘3’, and ‘4’ for XG, XF, XW, FJ, and ZG, respectively. All images were randomly divided into the training and testing sets in 8:2 ratio, that is, 392 images were utilized for the training set, and 98 images were used for the testing set. For the images of navel oranges, this study not only built a three-layer 2DCNN [58] but also applied three other popular deep neural networks in the field of machine vision (i.e., AlexNet [59], ResNet [60], and VGG11 [61]) to identify the origins of the navel oranges. The comparison of the origin traceability results of navel oranges based on NIRS and machine vision are shown in Table 5.

It can be known from Table 5 that the accuracy of thetesting set based on the image-based ResNet model was the lowest, merely 70.41%. The accuracy of the testing set based on the image-based 2DCNN model was less than 80%. Although the origin-traceability performance of navel oranges based on the image-based AlexNet and VGG11 was improved compared to the 2DCNN model, the accuracy of the testing set was still over 10% lower than that of the NIRS-based 1DCNN. Therefore, the origin-traceability performance of navel oranges based on RBG images was inferior to that of the NIRS combined with deep learning. Compared to the NIR spectra of navel oranges, the RGB images of navel oranges only contained the external information (color, size, texture, etc.) of navel oranges, but the external differences of Newhall navel oranges from different origins were not obvious. Even after training with deep learning models (2DCNN, AlexNet, ResNet, and VGG11), the feature information that could be extracted to reflect the origin difference was limited, which finally resulted in an unsatisfactory origin-traceability result of navel oranges based on RGB images. In contrast, NIR spectra could fully utilize the absorption characteristics of navel oranges to reflect deeper information related to the origin of navel oranges. Furthermore, based on deep learning algorithms, this part of the characteristic information could be extracted, thereby achieving a higher accuracy of origin traceability, as well as the precision, recall rate, and F1 score.

## 5. Conclusions

This study validated the feasibility of using NIRS combined with deep learning to trace the origin of navel oranges. The specific conclusions are given as follows: (1) A total of 490 Newhall navel oranges from five origins were selected to collect the raw NIR spectra via an FT-NIR spectrometer. The Mahalanobis distance method was used to eliminate abnormal samples, and SG+FD was determined as the optimal spectral preprocessing strategy for navel oranges based on the PLS-DA model. (2) A 1DCNN model was established with the optimal structure and parameters (three-layer convolution, kernel size = 3, batch size = 16, learning rate = 0.0001) to achieve the origin traceability of navel oranges. Compared with several machine learning models, the origin-traceability performance of the optimized 1DCNN model for the testing set was significantly better than that of other machine learning models, with an accuracy rate of 97.92%. (3) The effect of wavelength optimal selection methods on the origin traceability of navel oranges was discussed. The results showed that NIR spectra with no-wavelength optimal selection achieved the highest accuracy, and the computational time was the shortest. In addition, the origin-traceability results of navel oranges based on NIRS were compared with the machine vision method. The study found that the accuracy of origin traceability using NIRS combined with deep learning was superior to that of machine vision. Therefore, it was demonstrated that the combination of NIRS and deep learning enables rapid, non-destructive, and accurate geographical origin traceability of navel oranges, marking a significant advancement in the methods for origin traceability and quality control of navel oranges. Furthermore, this method can also provide powerful technical support for the classification and identification of the same samples with different spectra or the samples with the same color and morphology but different spectra.

## Figures and Tables

**Table 1:** The number of navel oranges sourced from each origin before and after removal.

Origin	Number of Samples Before Removal	Number of Samples After Removal
XG	100	95
XW	100	95
XF	100	99
ZG	94	93
FJ	96	96
Total	490	478

**Table 2:** Classification results of different preprocessing strategies based on PLS-DA models.

Preprocessing Strategy	PCs	Training Set Acc (%)	Testing Set Acc (%)
No-preprocess	10	81.41	79.16
SG	9	78.80	72.92
MSC	9	85.34	76.04
FD	6	84.03	81.25
SG+MSC	7	77.49	64.58
**SG+** **FD**	**8**	**89.79**	**84.38**
MSC+FD	6	86.13	77.08
SG+MSC+FD	6	84.82	80.21

**Table 3:** Comparison of model performance for the origin traceability of navel oranges between 1DCNN and PLS-DA, LDA, SVM, RF, and BPNN.

Model	Training Set *Acc* (%)	Testing Set				10-Fold CV *Acc* (%)
		*Acc* (%)	*P* (%)	*R* (%)	*F*1 (%)	
PLS-DA	89.79	84.38	84.38	84.6	84.37	78.43
LDA	77.75	69.79	70.45	69.44	69.11	72.34
SVM	85.08	73.96	78.78	74.30	73.98	73.20
RF	88.48	70.83	70.86	70.40	70.31	70.02
BPNN	78.01	77.08	86.08	75.71	70.37	80.40
**1DCNN**	**98.43**	**97.92**	**98.00**	**97.95**	**97.90**	**97.50**

**Table 4:** Comparison of 1DCNN modeling with different wavelength optimal selection algorithms.

Wavelength Selection Method	Number of Wavelengths	Training Set *Acc* (%)	Testing Set				10-Fold CV *Acc* (%)	Time (s)		
			*Acc* (%)	*P* (%)	*R* (%)	*F*1 (%)		Wavelength Selection Time	Modeling Time	Total Time
LAR	600	95.03	94.80	95.09	95.02	95.02	94.98	35.1	7.3	42.4
CARS	331	94.24	93.75	94.5	93.62	93.80	92.89	125.5	6.6	132.1
UVE	75	89.27	86.46	86.55	85.87	85.63	87.88	320.3	10.7	331
SPA	16	80.10	79.17	78.91	78.30	78.27	79.05	176.8	17.4	194.2
GA	677	96.60	95.83	96.16	96.20	96.16	95.82	2359.2	7.2	2366.4
**None**	**1556**	**98.43**	**97.92**	**98** **.00**	**97.95**	**97.9** **0**	**97.5** **0**	**0**	**8**	**8**

**Table 5:** The comparison of the origin traceability results of novel oranges based on NIRS and machine vision.

Data	Model	Training Set *Acc* (%)	Testing Set			
			*Acc* (%)	*P* (%)	*R* (%)	*F*1 (%)
**NIR Spectr** **a**	**1DCNN**	**98.43**	**97.92**	**98** **.00**	**97.95**	**97.9** **0**
RGB Image	2DCNN	89.79	76.53	79.54	77.12	76.40
	AlexNet	90.56	81.63	81.51	81.14	80.99
	ResNet	89.80	70.41	74.39	70.33	70.85
	VGG11	95.41	86.73	87.69	86.50	86.60

## Data Availability

All related data and methods are presented in this paper. Additional inquiries should be addressed to the corresponding author.
